# Book Reviews

**Published:** 1898-02

**Authors:** 


A Treatise on Gynecology, Medical and Surgical. By Dr. S. Pozzi, of Paris. Third, revised edition. Translated by Dr. Brooks H. Wells, of New York. One volume, of 950 pages, royal octavo, illustrated by over 600 wood engravings. New York : William Wood & Co. 1897.

The first American edition of this work was published in 1891 in two volumes, and received extended notice in the Journal, volume XXXI., new series, pp. 630 and 695. It was translated as is this edition by Dr. Wells, and was considered a decided contribution to the literature of gynecology. As an indication of the estimation placed upon Pozzi's treatise we may mention that it has been translated into the German, English, Spanish, Italian and Russian languages ; and it has also received the prizes of the Institute and the Academy of Medicine of Paris.

This is a translation of the third French edition, which has been thoroughly revised and to which many additions have been made. A few of the chapters, too, have been entirely rewritten. The subject of uterine fibroids, including their treatment by abdominal and vaginal hysterectomy, has been considered with especial care in the light of recent contributions to the subject. Pozzi's illustrations of fibromata and especially of the operations for their relief are particularly attractive as well as instructive.

All through the work are additions made by the American translator that contribute to the value of the text and serves to bring it forward to the latest period. These are included in brackets, making them readily distinguishable from the author's text. Some of the pathological anatomy is new and is illustrated by plates made from original drawings. notably that pertaining to ovarian cysts. We think the work has been greatly enhanced in value by the improvements we have noted and again by the fact that it is condensed into one volume.

The proficient teacher and practiser of gynecology must be in possession of literature from all parts of the globe, and especially of that of the French school, which has made important advances of late. No library, today, is complete that does not include Pozzi's treatise. It is beautifully printed and well dressed.

---

A Text-Book of Practical Therapeutics, with especial reference to the application of Remedial Measures to Disease and their Employment upon a Rational Basis. By Hobart Amory Hare, M. D., Professor of Therapeutics and Materia Medica in the Jefferson Medical College, Philadelphia, etc. With special chapters by Drs. George E. de Schweinitz, Edward Martin and Barton C. Hirst. Sixth edition, thoroughly revised and largely rewritten. In one octavo volume of 756 pages. Philadelphia and New York : Lea Brothers & Co., Publishers. 1897.

To write a book and prepare five more editions of it, six editions in all, within a period of seven years would keep most authors almost constantly occupied. But Dr. Hare has such a capacity for work, literary and clinical, that he has not only done this but has published many other treatises besides during the period mentioned. The remarkable feature of Dr. Hare's writings is that they contain well-digested facts or logical theories concisely stated and practically arranged. In other words, his books are not padded with verbosities and visionary propositions, but are written by a practical man and an accomplished teacher for practical physicians.

One of the most difficult problems in medicine, next in importance to diagnosis, is the application of remedies to the treatment of disease — practical therapeutics. It is oftentimes equally important to know when to withhold drugs, and this latter is one of the most perplexing lessons for the young doctor to learn. It would be well if every young physician would frame the motto that faces the title page of this book, original with the author, and hang it in a conspicuous place as a constant reminder of duty. It reads as follows : "When called to guide a patient through an illness the physician should be constantly a watchman, and a therapeutist only when necessity arises."

In preparing this sixth edition for the press the author has rewritten a considerable portion of the text and he has also arranged the work so as to make it useful in connection with his text-book of practical diagnosis, a notice of which appears coincidently with this one, though written more than a month previously.

The author has commendingly excluded from this treatise "remedies which are so rarely employed as to be only curiosities," and the space so saved is filled with details of the "application of well-tried remedies, both new and old."

There can be no two opinions over the fact that this is one of the best practical treatises on therapeutics extant and it should be in the hands of every student, clinician and family doctor.

An Epitome of the History of Medicine. By Roswell Park, A. M., M. D., Professor of Surgery in the Medical Department of the University of Buffalo, etc. Illustrated with portraits and other engravings. One volume, royal octavo, pages xiv.—348. Philadelphia: The F. A. Davis Co., Publishers, 1914 and 1916 Cherry street; New York: 117 W. Forty-second street; Chicago: 9 Lakeside Building.

It is essential for every intelligent physician to acquire a reasonable knowledge of the history of medicine. As a general rule the undergraduate has been in hot haste to obtain his degree and license that he may be enabled to proceed at once to the practice of his profession. He has been more interested in the commercial side of his profession than in its literary aspect—caring less for history than for shekels. For this condition of things pupils are not wholly to blame, but medical faculties must be made to share with them in the opprobrium. If the latter would take more pains to teach students something of accomplishments and help them to cultivate a taste for the esthetics of medicine, there would be less rudeness in their manners as they emerge from the lecture rooms, and more gentlemanly conduct in their bearing when they leave the halls of their alma mater and mingle with the world as physicians.

The work before us, which is the outgrowth of a course of lectures delivered by Dr. Park at the University of Buffalo, is a commendable effort in the direction that we have hinted at in the foregoing paragraph. Heretofore it has been difficult for a busy physician, and still more difficult for medical students, to obtain a concise historical account of medicine from the primitive period to our own times. It is one of the chief advantages of this book, and one that will readily command for it respect and a large audience, that it tells in pleasant form and attractive rhetoric the story of medical history from beginning to end.

The profession of medicine will be made better, stronger and cleaner by the possession and perusal of such books as this. It is refreshing to find in this period of haste, unrest and eager chase for gain such a book written by a busy doctor. It should be in possession of every physician, especially every young doctor. The book is well printed and otherwise presented in attractive form.

---

Practical Diagnosis. The Use of Symptoms in the Diagnosis of Disease. By Hobart Amory Hare, M. D., Professor of Therapeutics and Materia Medica in the Jefferson Medical College of Philadelphia; Laureate of the Medical Society of London, of the Royal Academy in Belgium, etc. New (second) and revised edition. In one octavo volume of 598 pages, with 201 engravings and thirteen full-page colored plates. Philadelphia: Lea Brothers & Co., Publishers. 1897.

The sudden exhaustion of the first edition of this unique treatise in a little more than a year from its appearance has rendered the publication of a second edition necessary. This has afforded the distinguished author an opportunity for revision, of which he gladly availed himself. It must be confessed, however, that the faults of the first edition were comparatively few: but in a progressive science even a year develops improvements, and such as have occurred are added in this book.

We devoted considerable space to a review notice of the first edition in our issue of January, 1897, Vol. XXXVI., No. 6, page 470. The particular object of this work was to become a guide at the bedside. That it fulfilled its mission is indicated by the fact that the profession is demanding the work even after it is out of press. An author must feel such a compliment markedly. So many books are printed that never reach a second edition—indeed, ought never to have seen the first—that it is refreshing to come across a book of this character. Hare is a voluminous writer, but he always writes to some purpose, never for the mere grouping of words. He is destined to become one of the foremost clinicians in the country.

The index of this book is remarkable, embracing, as it does, forty-six pages and giving symptoms as well as diseases, so that the searcher is sure to find what he needs. Every student, every teacher, every practitioner of medicine, should become familiar with this treatise.

---

TWELFTH ANNUAL REPORT OF THE STATE BOARD OF HEALTH and Vital Statistics of the Commonwealth of Pennsylvania. BENJAMIN B. LEE, M. D., Secretary. Volumes I. and II. 1896.

The Keystone state publishes a creditable report of its health department. In the annual report of the secretary to the board reference is made to the work of Dr. Lucien Howe, of Buffalo, in his endeavors to establish the Credé method for the prevention of blindness in infants.

The expenses of the conduct of the office during the year 1896 were about $4,000. How it is possible for a state health board to do efficient work with such a small expenditure is beyond our understanding. A state the size of Pennsylvania ought to place at the disposal of its board of health at least $100,000. The time has passed when the importance of health boards can be belittled. They play a most important function in the rôle of preventive medicine. The best talent that the state can command should be placed at its disposal and an adequate compensation should be paid to its officials.

Pennsylvania is, indeed, fortunate in obtaining so able and efficient a secretary as is Dr. Lee, and when we are told in this report that his traveling and office expenses for a year were only $1,886.56, we can but feel that his distinguished services are very meagerly paid for by the state.

In this volume Dr. Lee presents a very elaborate report relating to the meeting of the American Public Health Association at Buffalo in 1896. As we stated at the outset, this report is highly creditable in all its details.

